# Transcriptional and metabolic reprogramming induce an inflammatory phenotype in non-medullary thyroid carcinoma-induced macrophages

**DOI:** 10.1080/2162402X.2016.1229725

**Published:** 2016-09-09

**Authors:** Rob J. W. Arts, Theo S. Plantinga, Sander Tuit, Thomas Ulas, Bas Heinhuis, Marika Tesselaar, Yvette Sloot, Gosse J. Adema, Leo A. B. Joosten, Johannes W. A. Smit, Mihai G. Netea, Joachim L. Schultze, Romana T. Netea-Maier

**Affiliations:** a Department of Internal Medicine and Radboud Center for Infectious Diseases, Radboud University Medical Center, Nijmegen, the Netherlands; b Department of Pathology, Radboud University Medical Center, Nijmegen, the Netherlands; c Genomics and Immunoregulation, LIMES-Institute, University of Bonn, Bonn, Germany; d Department of Internal Medicine, Division of Endocrinology, Radboud University Medical Center, Nijmegen, the Netherlands; e Department of Tumor Immunology, Radboud Institute for Molecular Life Sciences, Radboud University Medical Center, Nijmegen, the Netherlands; f German Center for Neurodegenerative Diseases, Bonn, Germany

**Keywords:** Cytokines, epigenetics, immunometabolism, lactate, thyroid cancer, tumor-associated macrophages

## Abstract

Tumor-associated macrophages (TAMs) are key components of the tumor microenvironment in non-medullary thyroid cancer (TC), the most common endocrine malignancy. However, little is known regarding the regulation of their function in TC. Transcriptome analysis in a model of TC-induced macrophages identified increased inflammatory characteristics and rewiring of cell metabolism as key functional changes. This functional reprogramming was partly mediated by TC-derived lactate that induced upregulation of cytokine production through an AKT1/mTOR-dependent increase in aerobic glycolysis. This led to epigenetic modifications at the level of histone methylation, and subsequently long-term functional changes. Immunohistochemistry assessment validated the increase in glycolysis enzymes and lactate receptor in TAMs in tissue samples from patients with TC. In conclusion, Akt/mTOR-dependent glycolysis mediates TC-induced reprogramming of TAMs and inflammation, and this may represent a novel therapeutic target in TC.

## Abbreviations

BC-PAP: papillary TC, BRAF V600E mutation
CRA: co-regulation analysis
ECAR: extracellular acidification rate
FTC133: follicular TC, PTEN deficient
H3K4me3: histone 3 lysine 4 trimethylation
HIF1: hypoxia inducible factor
IFN: interferon
IL: interleukin
LPS: lipopolysaccharide
OCR: oxygen consumption rate
PBMC: peripheral blood mononuclear cell
RPMI: Roswell park memorial institute culture medium
SNP: single nucleotide polymorphism
TAM: tumor-associated macrophage
TC: thyroid cancer
TLR: Toll-like receptor
TNF: tumor necrosis factor
TPC1: papillary TC with a RET/ PTC rearrangement
TR: transcriptional regulator
VEGF: vascular epithelial growth factor

## Introduction

Tumor-associated macrophages (TAMs) and cancer cells have an important mutual relationship: on the one hand, the tumor modulates the function of infiltrating macrophages, and thus contributes to reprogramming of TAMs, while on the other hand TAMs influence the behavior of cancer cells.^1^ Theoretically, macrophages have the potential to play an effective role in elimination of tumor cells; however, there are also important lines of evidence that TAMs promote cancer cell survival, proliferation, metastases, angiogenesis, and immune suppression.^2^ The impact of TAMs on tumor progression very likely depends on their specific reprogramming within the tumor, a process influenced by factors of the local microenvironment such as hypoxia, release of local mediators (e.g., cytokines, growth factors), as well as metabolic products released by either the cancer cells and/or other immune and stroma cells.^1-4^ The pathways and mechanisms involved in the reprogramming of TAMs are incompletely understood and in the present study, we aimed to investigate the phenotypic and functional characteristics of TAMs in a model of non-medullary thyroid cancer (TC). TC is the most common endocrine malignant tumor and of all cancer types has the highest rise in incidence.^5^ Previous studies have shown that particularly anaplastic and poorly differentiated TCs, which have a very poor prognosis, are highly infiltrated with TAMs and that this correlates with prognosis. In these tumors, TAMs represent the most prominent component of the tumor microenvironment.^6,7^ Pharmacological depletion of TAMs using CSF-1R inhibition in a murine model of TC has been reported to reduce tumor development and progression.^8^ These data suggest that TAMs have pro-tumorigenic effects in TC and might represent promising therapeutic targets for tumors that are resistant to conventional treatment. Nevertheless, little is known about on the role of the TAMs in TC and on the factors driving the functional reprograming the macrophages within the context of TC. To this end, we used a complementary approach that combines a discovery-based methodology on transcriptome analysis of human monocytes that have been co-cultured with TC cells and differentiated into TC-induced macrophages to reveal essential pathways in specific reprogramming of these macrophages, with immunological, metabolic, and genetic approaches to validate the pathways identified. We show that TC-induced macrophages are characterized by important immunological and metabolic rewiring that impacts their function and that these metabolic changes are being recapitulated in the TAMs in tissue samples from patients with TC. These findings may help the identification of novel therapeutic approaches for TC and potentially for other malignant processes as well.

## Results

### Transcriptome analysis: Inflammatory and metabolic rewiring in TC-induced human macrophages

**Figure 1.**
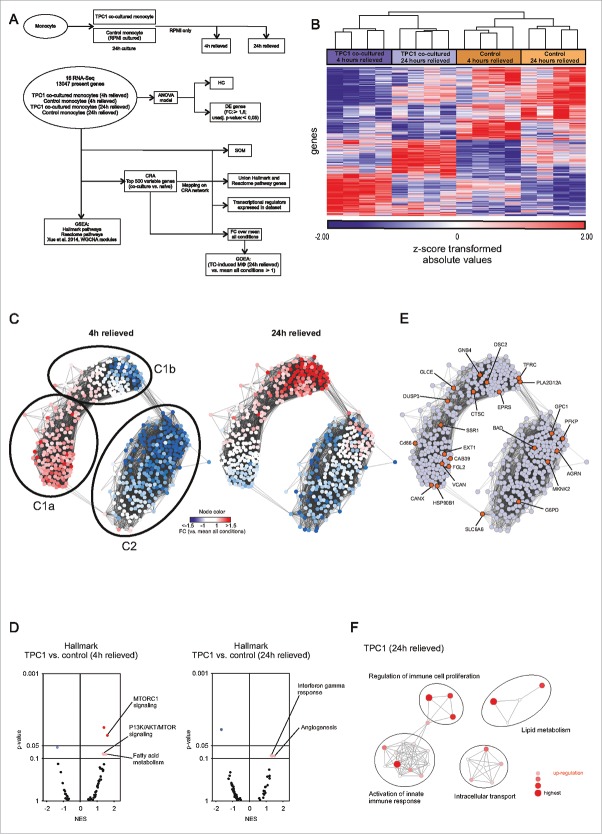
Transcriptome analysis of TC-induced macrophages. (A) Scheme outlining the experimental setup (upper scheme) and bioinformatics analysis (lower scheme). (B) HC map of the top 1,000 variable genes between TC-induced macrophages and RPMI controls. (C) Network visualization of gene-centered CRA (Pearson correlation ≥ 0.7). FC over mean of all conditions is mapped onto the network for TC-induced macrophages (4 h, left network; 24 h, right network). (D) Volcano plots of normalized enrichment scores (NES) and enrichment p-values based on GSEA using Hallmark pathway gene sets (MSigDB, Broad Institute). Data are shown for TPC1 co-cultured macrophages (4 h and 24 h). Dark (NES ≥ 1; *p*-value ≤ 0.05) and light (NES ≥ 1; *p*-value > 0.05, ≤ 0.1) red circles show gene sets positively enriched. Dark and light blue circles show gene sets depleted (NES ≤ −1). (E) Hallmark and reactome pathway genes (MSigDB, Broad institute) related to PI3K/AKT/MTOR and glycolysis mapped onto the CRA network (orange nodes). (F) Network visualization of GOEA of genes derived from the “TPC1 co-cultured (24 h) vs. mean all conditions” comparison (FC: ≥ 1; present in CRA network) using BiNGO and EnrichmentMap. Red nodes represent the enriched GO-terms, node size and color represents corresponding FDR-adjusted enrichment *p*-values (*p*-value ≤ 0.05). See also Fig. S1.

To enable a detailed analysis of the effect of TC cells on monocyte reprogramming, we generated genome-wide transcriptome data by RNA-sequencing of TC-induced macrophages and RPMI cultured control monocytes (24 h of culture), both of which were relieved for either 4 h or 24 h in RPMI after co-culture with the tumor cells (TPC1 cell line). We performed an extensive bioinformatics analysis as outlined in (Fig. 1A). A total of 13,047 transcripts were identified as present throughout 4 conditions represented by 16 samples. Employing a two-way ANOVA model (Table S1), we identified 372 (4 h) and 353 (24 h) genes being differentially expressed between TC-induced macrophages and RPMI controls (Fig. S1A). Next, we visualized the degree of variance between TPC1 co-cultured monocytes and control cells at both time points by performing hierarchical clustering (HC) based on the top 1,000 variable genes (Fig. 1B). HC revealed that TPC1 co-cultured monocytes exert significant transcriptomic changes compared to control monocytes. Moreover, there was also a clear difference in gene expression between monocytes co-cultured with TPC1 for 4 h, respectively, 24 h, suggesting that the observed changes are dynamic. To validate these findings, we applied co-regulation analysis (CRA) and visualized the gene network of the top 500 variable genes within the data (Fig. 1C). The topology of the network was defined by two major clusters (C1, C2), with cluster C1 showing a clear substructure (C1a, C1b). Overlaying differentially regulated genes onto the network revealed that the cluster C1a represented genes increased in expression in monocytes co-cultured with TPC1 at the 4 h time point, while C1b was associated with genes elevated at the 24 h time point. Additionally, employing Self-Organizing Map (SOM) clustering to identify and visualize conditions-specific genes within the network (Fig. S1B), we were able to validate the condition-specific sub-networks. Together, these data support a model suggesting transcriptional programming of monocytes by TPC1 tumor cells in a dynamic fashion. To investigate the biological relevance of the observed transcriptomic changes in both 4 h and 24 h TC-induced macrophages, we applied Gene Set Enrichment Analysis (GSEA) using two well-established pathway gene sets.^9^ Computing enrichment for hallmark pathways (Fig. 1D), “PI3K/AKT/MTOR,” and “fatty acid metabolism” pathways were enriched at the 4 h time point. In contrast, the TPC1 co-cultured monocytes taken at the 24 h time point showed enrichment of “interferon gamma response” and “angiogenesis” related pathways. Using reactome pathways to compute enrichment, we detected even more inflammation-related pathways to be enriched at the 24 h time point (e.g., inflammasome activation, TLR responses, interferon signaling, etc.) (Fig. S1C, Table S2). It is known that cellular metabolism of macrophages is crucial for inflammatory function, with a shift from oxidative phosphorylation toward mTOR-dependent aerobic glycolysis playing a central role.^10,11^ Therefore, we extracted the gene sets from PI3K/AKT/mTOR- and glycolysis-related pathways (both hallmark and reactome) and mapped them onto the CRA network (Fig. 1E). Indeed 16 of 23 genes were found in cluster C1 (Fig. 1E) and were elevated at the 4 h (12/16) and 24 h (10/16) time points, respectively (Table S3). To determine whether potential transcriptional regulators (TR) of mTOR-dependent aerobic glycolysis might also be part of the network, we mapped TR information onto the CRA network (Fig. S1D). Surprisingly, 23 TRs were downregulated (cluster C2) in TPC1 co-cultured monocytes, while only seven TRs were upregulated. Indeed, among the upregulated transcription factors, we identified hypoxia-inducible factor-1 α (HIF1A, orange node), which was previously shown to be involved in mTOR-dependent aerobic glycolysis ^10^ in cluster C1a. RelA, a NF-kB transactivating subunit that has been shown to counteract reprogramming to aerobic glycolysis ^12^ was found to be downregulated in TPC1 co-cultured monocytes. Moreover, the reactome pathway “NF-kB is activated and signals survival” was found to be depleted in TPC1 co-cultured monocytes at the 4 h time point (Fig. S1C). We next validated the findings for the 24 h time point employing Gene Ontology Enrichment Analysis (GOEA) on genes elevated in TPC1 co-cultured monocytes (fold change (FC) over mean: ≥ 1, present in the CRA network). Network visualization of enriched GO-Terms revealed terms related to “immune cell proliferation” and “activation of innate immune response” (Fig. 1F) further supporting reprogramming of monocytes at the later time points toward immune activation. Taken together, our data analysis supported a model of transcriptional regulation of genes involved in mTOR-dependent glycolysis early after exposure to TC cells, while further reprogramming toward an inflammatory response was seen at the later time point.

### TC-induced macrophages are reprogrammed by the secretome of TC cells

**Figure 2.**
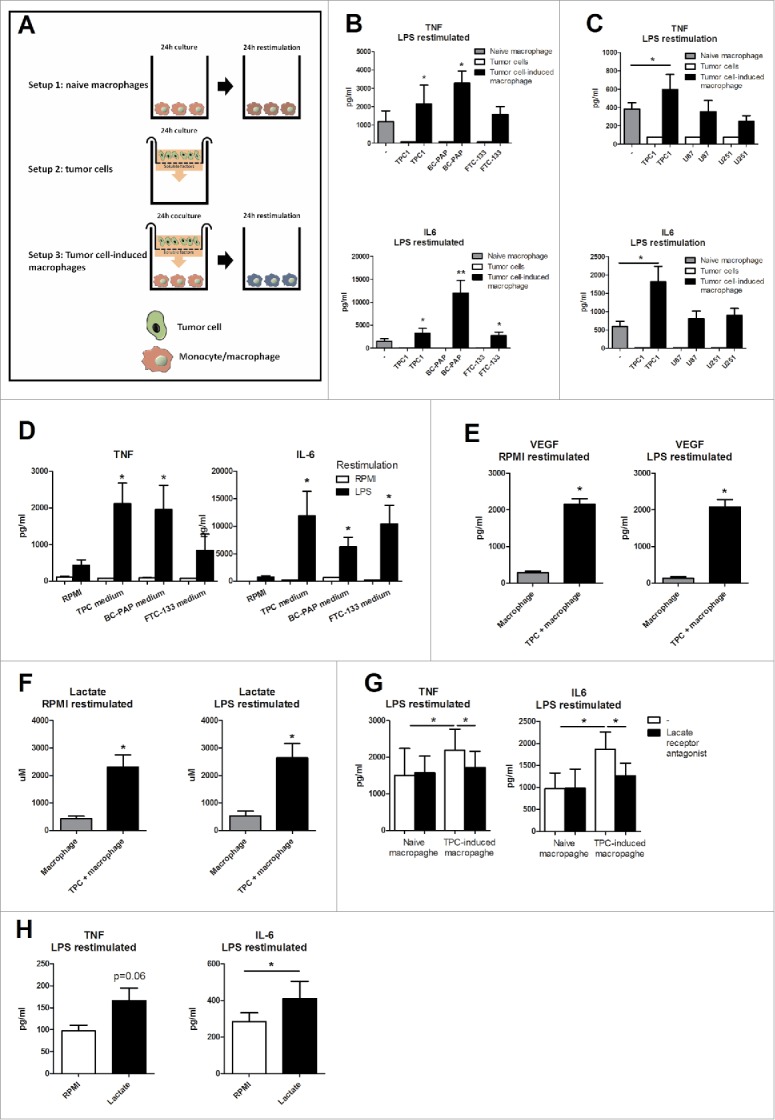
TC-induced macrophages have a proinflammatory phenotype. (A) Outline of the culturing methods. (B) Monocytes were incubated for 24 h with TC cells in a trans-well system and restimulated with LPS for 24 h (n = 7). (C) TAMs derived from co-culture with TPC1 and two glioblastoma cell lines were restimulated with LPS for 24 h (n = 4). (D) Medium from three thyroid cell lines was added to human monocytes for 24 h and subsequently stimulated with RPMI or LPS for 24 h (n = 4). (E & F) Lactate and VEGF were determined in naïve macrophage and tumor medium (n = 6). (G) A lactate receptor antagonist was added to the culture system. (Mean ± SEM, n = 5, *p < 0.05, by Wilcoxon signed-rank test). (H) Monocytes were incubated with 1 µM of lactic acid for 24 h and restimulated with LPS for 24 h (n = 8). Data shown as Mean ± SEM, *p < 0.05, **p < 0.01 by Wilcoxon signed-rank test.

Transcriptome analysis suggested a proinflammatory profile of TC-induced macrophages. We aimed to validate this finding by functional assessments: human primary monocytes were co-incubated in a trans-well system with the three types of TC cell lines (TPC1, FTC133, and BC-PAP) for 24 h, followed by restimulation for 24 h with either culture medium (RPMI), or Toll-like receptor (TLR) ligands (TLR4 ligand lipopolysaccharide (LPS), TLR2 ligand (Pam3CKS4 (P3C), or IL-1α) (Fig. 2A). The production of TNF and IL-6 by TC-induced macrophages was significantly increased compared to the control naïve macrophages (Fig. 2B and Fig. S2A). Production of IL-10 and IL-1β was low and in most conditions below detection limit. Additional experiments using TPC1-induced macrophages showed also increased IL-8 production, whereas no differences in granulocyte macrophage colony-stimulating factor (GM-CSF), monocyte chemotactic protein 1 (MCP1) were found (Fig. S2B). Co-culture of human monocytes with two glioblastoma cell lines showed less strongly upregulated cytokine production upon restimulation, suggesting a degree of specificity for the effect of the TC cell lines (Fig. 2C).

The identification of the proinflammatory profile induced by TC cells in monocytes lead to the hypothesis that soluble factors released by TC cells impact on the differentiation of these monocytes into macrophages with a specific phenotype. To reveal whether a soluble factor in the medium produced by the cancer cells was responsible for the upregulated cytokine production, medium from TC cell line cultures was added to human monocytes for 24 h, after which cells were restimulated with LPS. In line with previous experiments, this increased production of cytokines from macrophages as well (Fig. 2D). Two factors known to be released by tumor cells and which have immunologic effects are vascular epithelial growth factor (VEGF) and lactate, the end-metabolite of glycolysis. Indeed, both VEGF (Fig. 2E) and lactate (Fig. 2F) concentrations were significantly increased in TC conditioned media. In order to determine whether lactate or VEGF could serve as soluble factors that are necessary for the specific reprogramming of the TC-induced macrophages, antagonists of the cellular receptors for these molecules were added to the culture system. Blockade of lactate receptor significantly reduced cytokine release by TC-induced macrophages, while blockade of the VEGF receptor had no effects on cytokine release (Fig. 2G and Fig. S3). These data suggest that TC cell-derived lactate contributed to the induction of the inflammatory profile of TC-induced macrophages. In line with this, preincubation for 24 h with 1 µM of lactate also increased cytokine production upon TLR stimulation (Fig. 2H).

### TC-induced macrophages display increased glucose metabolism that is necessary for increased cytokine production

**Figure 3.**
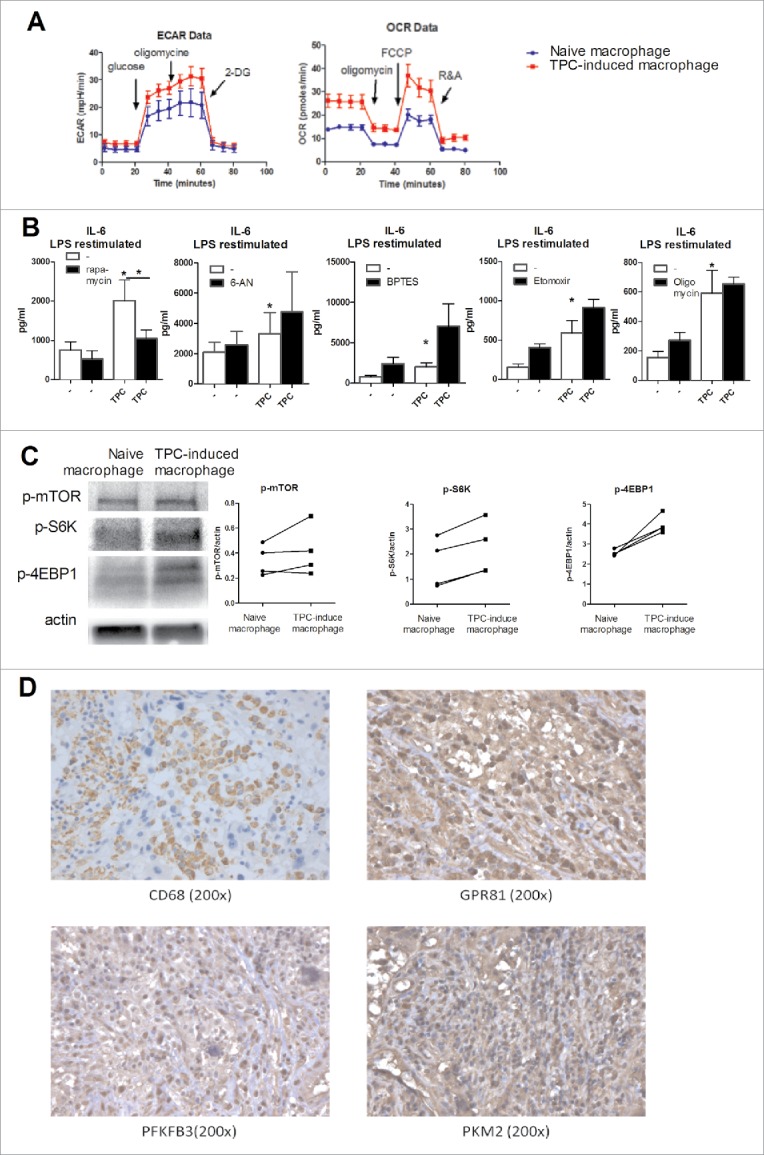
Metabolism of TC-induced macrophages is changed. (A) Extracellular acidification rate (ECAR) and oxygen consumption rate (OCR) from TC-induced macrophages and naive macrophages were determined after TC-induced macrophages were relieved for 24 h from the TPC1 cells (n = 4). (B) Inhibitors of mTOR (rapamycin), pentose phosphate pathway (6-AN), glutamine metabolism (BPTES), fatty acid β oxidation (etomoxir), and complex V ATP synthase (oligomycin) were added to the culture system and cells were restimulated for 24 h with LPS (n = 4). (C) Monocytes were incubated for 24 h with TPC1 cells, after 24 h rest cells were lysed and p-mTOR, p-S6K, and p-4EBP1 induction were determined. (D) Immunohistochemical analysis of PFKFB3, PKM2, and GPR81 in CD68-positive TAMs. Results are representative of stained FFPE tissue material from six anaplastic TC patients. Data shown as Mean ± SEM, *p < 0.05, by Wilcoxon signed-rank test.

Transcriptome analysis of TC-induced macrophages also revealed that several metabolic pathways were upregulated at transcriptional level. We and others have shown that cellular metabolism of macrophages is crucial for their inflammatory function, with a shift from oxidative phosphorylation toward mTOR-dependent aerobic glycolysis (Warburg effect) playing a central role.^10,11^ To investigate the activation of glycolysis and oxidative phosphorylation in TC-induced macrophages, extracellular acidification rate (ECAR), and oxygen consumption rate (OCR) of TC-induced macrophages (before restimulation by TLR engagement) were measured by Seahorse technology. Interestingly, maximal ECAR was increased in TC-induced macrophages and OCR was increased at both basal and maximal level (Fig. 3A). The intracellular concentration of acetyl CoA was increased, which could be used both to fuel the TCA cycle and for fatty acid synthesis. The glutamate concentration was decreased, likely caused by the replenishment of the TCA cycle through glutamine metabolism (Fig. S4). These data demonstrate strong activation of metabolic activity in the TC-induced macrophages.

To assess which of the metabolic pathways is essential in the upregulation of cytokine production, specific metabolic pathways were inhibited and cytokine production was assessed. Inhibition of the pentose phosphate pathway (6-aminonicotinamide, 6-AN), glutamine metabolism (BPTES), β oxidation of fatty acids (etomoxir), or the electron transport chain complex V (oligomycin) did not influence cytokine production. However, exposure to mTOR inhibitor rapamycin significantly decreased IL-6 production by TC-induced macrophages, demonstrating a role for mTOR/glycolysis pathway in this process (Fig. 3B and Fig. S5). This was further supported by assessing phosphorylation of mTOR and its downstream products (S6K and 4EBP1) in TC-induced macrophages before restimulation. Indeed, we observed enhanced activation of this pathway in TC-induced macrophages (Fig. 3C).

In order to validate activation of glycolysis in TC-derived TAMs in patients with TC, formalin-fixed paraffin-embedded (FFPE) tissue sections of six thyroid tumors were immunohistochemically prepared and TAMs were stained by CD68 staining. To investigate the extent of glycolysis in these TAMs, expression of the third human isoform of 6-phosphofructo-2-kinase/fructose-2,6-bisphosphatase and pyruvate kinase 2 (PFKFB3, PKM2), and the lactate receptor GRP81 were determined. Both these key enzymes of glycolysis and the expression of the lactate receptor were highly upregulated in at least 80% of CD68-positive TC-associated macrophages present in these tissues. (Fig. 3D and Fig. S6A). In contrast, virtually no macrophages could be detected in normal thyroid tissues from the same individuals (Fig. S6B).

### TC-induced macrophages undergo epigenetic reprogramming regulated by the mTOR pathway

**Figure 4.**
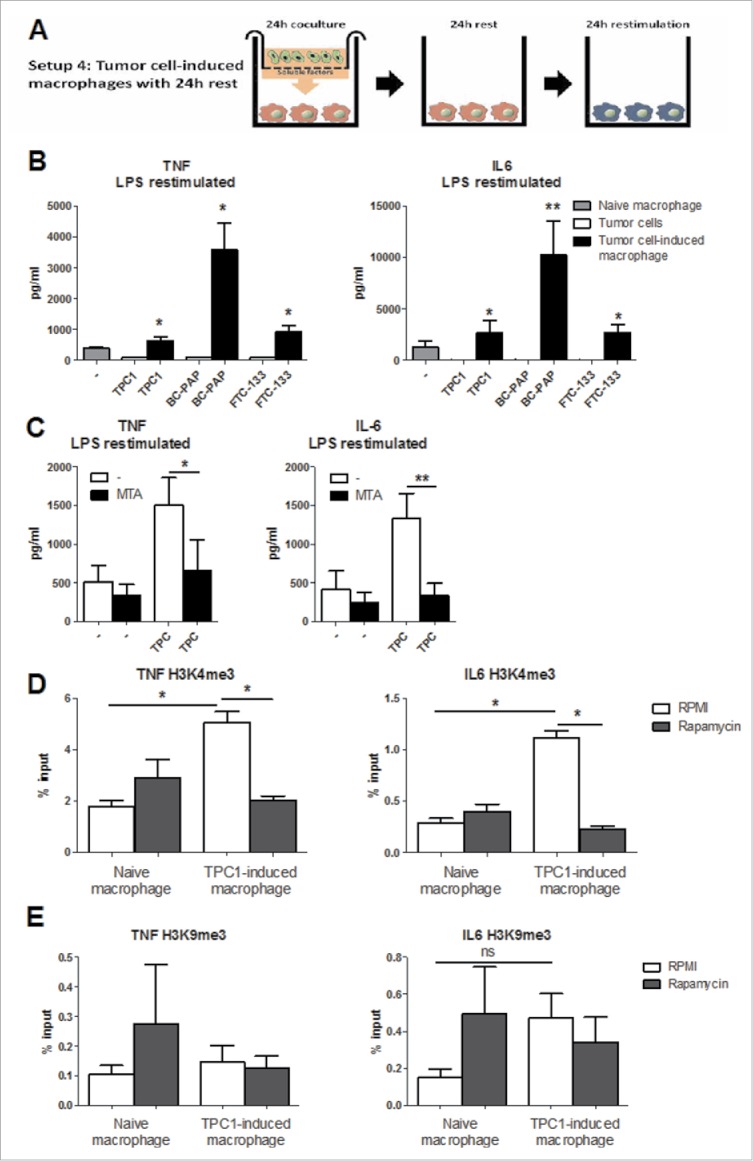
The proinflammatory phenotype is epigenetically regulated. (A) Outline of the culturing methods. (B) Monocytes were co-incubated with thyroid tumor cell lines for 24 h. After 1 day additional rest they were restimulated with LPS for 24 h (n = 5). (C) MTA, an inhibitor of histone methylation was added to the culture system and cells were restimulated for 24 h with LPS (n = 6). (D & E) Monocytes were incubated for 24 h with TPC1 cells, after 24 h rest DNA was isolated to determine H3K4me3 (D) and H3K9me3 (E) expression at the promoter site of *IL6* and *TNFA* (n = 6, n = 5). Data shown as Mean ± SEM, *p < 0.05 by Wilcoxon signed-rank test.

In the following set of experiments, we wanted to assess whether TC cell-macrophage co-incubation induced a stable inflammatory phenotype over time. To assess this, after the initial preincubation with TPC1 cells, the TC-induced macrophages were left in culture medium for an additional 24 h before restimulation (Fig. 4A). The restimulation with LPS showed that the cytokine production was still upregulated in the TC-induced macrophages even if they were relieved from the TPC1 cells, demonstrating a long-term stable phenotype (Fig. 4B). This suggests a putative epigenetic rewiring of the transcriptional program, as shown in other types of long-term functional reprogramming of macrophages, for example, during trained immunity.^13^ In order to test this hypothesis, a broad histone methylation inhibitor (MTA) was added during the first 24 h of co-culture. This brought cytokine production back to baseline (Fig. 4C), showing that histone methylation plays an important role in the specific reprogramming of TC-induced macrophages. H3K4me3 is an important histone marker of open (accessible) promoters shown to be associated with high cytokine production.^14^ To assess whether increased H3K4me3 plays a role in the long-term inflammatory phenotype of TC-associated macrophages, monocytes were preincubated for 24 h with TPC1 cells, after which the tumor cells were removed and the macrophages were incubated for an additional 24 h in culture medium. Chromatin immunoprecipitation was performed to determine the H3K4me3 status of the promoters of *IL6* and *TNFA* in TC-induced macrophages. Indeed, a significant increase of H3K4me3 was observed in the TC-induced macrophages. Additionally, when rapamycin was added to the culture medium, the increase of H3K4me3 was abolished, which corresponded to the observed decrease of IL-6 after mTOR inhibition with rapamycin (Fig. 4D). This suggested that the induction of the mTOR-dependent pathway plays an important role for long-term epigenetic reprogramming of TC-induced macrophages. H3K9me3 is a histone mark that induces heterochromatin and therefore decreases DNA accessibility^14^, which was also found to be important in trained immunity.^15^ A potential role of H3K9me3 was assessed, but no significant downregulation of H3K9me3 was found after co-culture with TPC1 tumor cells (Fig. 4E).

## Discussion

The process through which monocytes infiltrate tumors, differentiate into TAMs, and influence the immune responses toward the malignant cells is believed to play a crucial role in carcinogenesis. TC is the most common endocrine malignancy, and TAMs have been shown to be strongly present in the tumor and impact the prognosis.^6,7^ However, despite this, very little is known about the phenotype of TAMs in TC and the pathways that influence macrophage differentiation in TC. In the present study, we initiated transcriptome analysis of TC-induced macrophages, and we demonstrate that soluble factors released by TC (among which lactate) induce a strong inflammatory phenotype and a rewiring of the cellular metabolism of TC-induced macrophages. Activation of mTOR-dependent glycolysis induced long-term epigenetic histone modifications (especially H3K4me3) and upregulated cytokine production. The role of the lactate-glycolysis-inflammation circuit for TC was also further supported in patients through immunohistological and genetic approaches.

The assessment of the transcriptome of the TC-induced macrophages identified complex circuits of immunological and metabolic pathways. One of the strongest set of cellular functions highly upregulated in the TC-induced macrophages were those related to inflammation: pathways related to pattern-recognition receptors, chemokine synthesis, and cytokine–cytokine receptor interaction were upregulated, suggesting a strong inflammatory phenotype of the cells. This was validated by experiments demonstrating that macrophages differentiated in the presence of TC cell lines displayed much stronger inflammatory function upon stimulation. An inflammatory phenotype has been demonstrated to characterize TAMs in many tumors^16^, and inflammation stimulates carcinogenesis through multiple pathways: excretion of epidermal growth factor, proangiogenic signals such as VEGF and fibroblast growth factor 2, several proinflammatory cytokines and chemokines, and factors such as matrix-degrading enzymes, metalloproteinases, cysteine cathepsin proteases, and heparanases all contribute to a tumor-promoting environment.^17,18^ These data strongly suggest that TAMs-derived inflammation has an important impact on TC development. Indeed, it has been previously shown that the presence of inflammatory mediators in TC, including cytokines such as IL-1β and TNFα, negatively influence the expression of sodium iodine symporter, the major protein responsible for the uptake of radioactive iodide in thyroid cells which represents the main targeted therapy for patients with TC.^19^ Also processes that limit inflammation such as autophagy are defective in TC.^20,21^ In addition to TAMs, also the function of regulatory T cells has been shown to be dependent on glycolysis, as the induction of their suppressive function was tightly dependent on mTOR-induced glycolysis.^22^ This is especially interesting as more aggressive forms of TC are associated with increased numbers of regulatory T cells in peripheral blood ^23^ and TAMs are able to induce regulatory T cells.^24,25^ The interaction and balance between the proinflammatory macrophages and inhibitory regulatory T cells, whose activity is both dependent on glycolysis, might therefore be an interesting topic for future research.

In addition to the inflammatory pathways induced by TC in TAMs, a second important set of biological processes strongly modulated were the metabolic pathways, with strong upregulation of the mTOR signaling pathway, carbohydrate digestion, but also fatty acid and pyrimidine biosynthesis. Changes in cellular metabolism, and especially a shift toward aerobic glycolysis through mTOR-dependent mechanisms, have been recently demonstrated to be crucial for the activation of macrophages.^10,11^ Moreover, inflammatory (M(IFNγ), formely M1) macrophage metabolism is associated with an increased glucose consumption and lactate production, whereas the more anti-inflammatory (M(IL4), formally M2) macrophages depend preferentially on oxidative phosphorylation. On the other hand, M(IL-4) macrophages typically express more arginase, which is necessary in amino acid metabolism^26,27^ and M(IL-4) macrophages also show more fatty acid uptake and β-oxidation compared to M(IFNγ) macrophages.^28^ Interestingly, the transcriptome and functional phenotype of TC-induced macrophages did not display a clear M(IFNγ) or M(IL-4) phenotype, with both pathway characteristics of M(IFNγ) (inflammation, glycolysis) and M(IL-4) (oxidative phosphorylation) being upregulated. Interestingly, when we compared our results to the transcriptome of a panel of 29 different macrophage profiles as reported by Xue *et al*.^29^, the transcriptional program of 4 h TC-induced macrophages was defined by gene modules associated with macrophage stimulation by fatty acids (linoleic, oleic, lauric acid), LPS or a combination of TNF, prostaglandin E2 and Pam2Cys (TPP). The transcriptional program of 24 h TC-induced macrophages was mainly described by IL-4 defined gene modules in combination with an inflammatory gene module linked to stimulation with palmitic acid (PA) (Fig. S1E). Therefore, this mixed gene signature in TC-induced macrophages cannot be considered as an archetypical M2 macrophage. We thus propose that the functional program induced by TC in TAMs is distinct and combines elements from both M(IL-4) macrophages, and inflammatory characteristics. In addition to IL-4, another important anti-inflammatory cytokine is IL-37, which could be especially relevant due to its capacity to inhibit the Akt/mTOR pathway.^30,31^ While no IL-37 expression could be found in the TC-induced macrophages, future studies should investigate it in *in vivo* samples from TC patients.

One of the most relevant findings of metabolic pathways activated in TC-induced macrophages is mTOR-dependent glycolysis. Indeed, we demonstrate an essential role for the AKT/mTOR pathway for the inflammatory phenotype of the TC-induced macrophages and this could represent an important therapeutic target. In TAMs induced by other tumors it has been shown that lactic acid produced by the cancer cells is essential for TAM reprogramming, by induction of the transcription factor HIF-1α^3^, and a similar dependency on lactate release was observed for the cytokines released in our system. As HIF-1α activation is highly dependent on mTOR activation, our observation that HIF-1α is one of the main TFs in TC-induced macrophages fits with this picture. Interestingly, inhibiting mTOR with rapamycin abolished IL-6 upregulation induced by TC cells, while TNFα production was not affected. In addition, mTOR inhibition was accompanied by downregulation of the histone marks associated with open chromatin and increased transcription.

**Figure 5.**
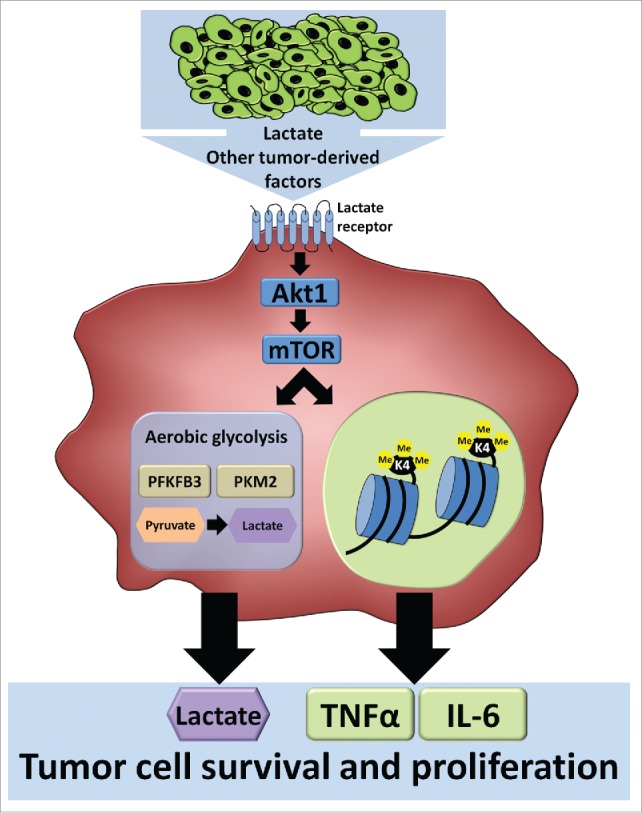
Soluble tumor-derived signals change the phenotype of the tumor-associated macrophage, by induction of the mTOR pathway and glycolysis, and epigenetic changes.

This role of the mTOR pathway provides a link between cellular metabolism and epigenetic programming of gene transcription. The field of metabolo-epigenomics is an emerging field within immunology ^32^, with important roles demonstrated for various metabolites (especially of glycolysis and TCA cycle) in modulating epigenetic processes. For examples, acetyl-CoA influences histone acetylation by functioning as a substrate for histone acetylation, succinate and fumarate inhibit histone demethylases, and NAD^+^ induces histone deacetylation through activation of sirtuins.^32-34^ Recently, the rate of glycolysis was directly associated with the acetylation of specific histone sites, which appeared to be mostly dependent on acetyl-CoA levels.^35^ We show an upregulation of glycolysis, and an increased intracellular concentration of acetyl Co-A in TC-induced macrophages. Moreover, glycolysis has been shown to be a source (together with folic acid and methionine) for S-adenosylmethionine (SAM), which serves as a methyl donor for histone and DNA methylation.^33^ This is especially interesting because of increased TCA cycle metabolism and oxidative phosphorylation in TC-induced macrophages. All in all, these data strongly suggest that the metabolic rewiring induced in macrophages by TC-derived compounds (such as lactate) induces epigenetic reprogramming (as demonstrated by enhanced H3K4me3), which in turn results in increased gene transcription of inflammatory mediators (see scheme Fig. 5). We hypothesize that this enhanced inflammation will in turn influence tumor progression and outcome.

In addition to glycolysis, other metabolic pathways were also modulated in TC-induced macrophages. Interestingly, inhibition of glutamine metabolism that can contribute to replenishment of the TCA cycle did not reduce cytokine production in TAMs, but showed a tendency toward increased cytokine production. This is an interesting observation, which has not been further investigated, but should be addressed in future research. In addition, fatty acid synthesis was very strongly increased in TC-induced macrophages: although an effect on inflammation and cytokine production was not observed, this may very well impact other aspects of tumor and macrophage biology such as cell survival and proliferation.

Our observation that reprogramming of tumor stroma cells (in our case TAMs) is associated with changes in metabolism is reminiscent of a process termed “reverse Warburg effect.”^36,37^ In this process it has been suggested that cancer cells reprogram metabolism of tumor stroma cells toward aerobic glycolysis (Warburg effect), and this will result in fuel for mitochondrial metabolism (e.g., lactate and ketones), which is beneficial for tumor growth and survival. Using oxidative phosphorylation instead of glycolysis reduces the speed of cell aging and is therefore beneficial for tumor cell survival, growth, and metastasis.^37^ Whether these changes are a result of local reprogramming of myeloid cells in the tumor microenvironment or whether soluble factors excreted by the tumor (stroma) already reprogram circulating monocytes, or even the myeloid precursors in the bone marrow, remains unanswered and should be investigated in future studies.

In conclusion, we show that TC-induced macrophages display broad changes in cellular metabolism and have an increased inflammatory profile. Reprogramming of the TC-induced macrophages is dependent on activation of the AKT/mTOR pathway, which is also essential for reshaping the epigenetic landscape.

## Materials and methods

### Co-culture model of TC cell lines and human monocytes

The TC cell line *in vitro* experiments were performed using TPC1 (papillary, RET/ PTC rearrangement), BC-PAP (papillary, BRAF V600E mutation), and FTC133 (follicular, PTEN deficient) cell lines.^38^ The human glioblastoma cell lines U87 and U251 were kindly provided by Prof. G.J. Adema and Dr P. Gielen, Radboudumc Nijmegen. Peripheral blood mononuclear cells were isolated by density gradient centrifugation using Ficoll-plaque (GE Healthcare, Diegem Belgium) from buffy coats obtained from Sanquin bloodbank, Nijmegen, The Netherlands, or healthy volunteers after informed consent (ethical approval CMO 2010-104). After adhesion for 1 h in the 96-well plates, the non-adherent cells comprising mainly lymphocytes were discarded, and the adherent monocytes were incubated further with the tumor cell lines. In selected experiments such as those assessing cell transcriptome, an additional step of purification using CD14-labeled magnetic beads was performed in order to obtain a highly purified monocyte population.

The cancer cell lines were grown in culture medium RPMI 1640 Dutch modification (Life Technologies, Carlsbad, California, USA) supplemented with gentamycin 50 µg/mL, pyruvate 1 mM, glutamax 2 mM and 10% Fetal Calf Serum (Gibco, Life Technologies). A trans-well system used ThinCert^TM^ cell culture inserts on a 24-well plate (Greiner Bio-One GmbH, Austria). Cell counts were performed in a Coulter particle counter (Beckman Coulter Inc.). A total of 1.0 × 10^5^ TPC1 cells (or the other TC cell lines, as indicated) in 250 µL culture medium were added to the upper compartment of the trans-well system, while RPMI was added to the lower compartment. The cells were incubated for 24 h at 37°C, 5% CO_2_. Next, the cells were washed with Phosphate Buffered Saline (PBS, Braun Melsungen, Germany) and PBMCs were prepared in culture medium: RPMI (Life Technologies, Carlsbad, California, USA) supplemented with glucose 5 mM, pyruvate 1 mM, glutamine 2 mM, gentamicin 50 µg/mL and HEPES 10 mM (Life Technologies, Carlsbad, California, USA). A total of 1.0 × 10^6^ PBMCs in 500 µL were added to the lower compartment of the trans-well system. PBMCs and cancer cell lines were co-cultured for 24 h at 37°C. In some experiments, the mTOR inhibitor rapamycin (100 nM, Bio Connect, Huissen, The Netherlands), glutaminase inhibitor BPTES (50 µM, Sigma – Aldrich Chemie, Zwijndrecht, The Netherlands,), G6PD inhibitor 6-aminonicotinamide (100 nM, Sigma), CPT-1 inhibitor etomoxir (10 µM, Sigma), ATP synthase inhibitor oligomycin (1 µM, Sigma), 500 µm 5′-deoxy-5′(methylthio) adenosine (MTA, sigma), lactate receptor antagonist α-cyano-4-hydrocycinnamioic acid (1 mM, Sigma) or VEGF receptor antagonist Sorafenib (100 nM, Bio Connect) were added to the medium.

After 24 h incubation, the cell culture inserts containing RPMI control medium or TPC1 cells were discarded and the PBMCs were stimulated for 24 h with RPMI or 10 ng/mL LPS (*E. coli* strain O55:B5, Sigma Chemical Co, St. Louis, MO), as substitute for endogenous TLR4 ligand signaling, 10 µg/mL Pam3Cys (EMC microcollection) or 10 ng/mL IL-1α (Hoffman/Roche). At the end of the incubation period, supernatant was collected and stored at −20°C until further analysis, or cells were collected for further analysis.

### RNA isolation followed by deep sequencing

Total RNA was extracted from 24 h TPC1 co-cultured macrophages and 24 h RPMI cultured monocyte controls (four donors) after 4 h and 24 h of additional incubation in RPMI only, using RNeasy Mini and Microkits (Qiagen). 50 ng of RNA was converted into cDNA libraries according to the TruSeq RNA library preparation kit v2 and libraries were sequenced on the HiSeq 1500 system and demultiplexed using CASAVA v1.8 (Illumina). Detail of the further analysis can be found in the Supplementary data. The detailed data have been deposited in the GEO database with accession number GSE76445.

### Cytokine, chemokine, VEGF, and lactate production

Interleukin 6 (IL-6), IL-8 (Sanquin, Amsterdam, Netherlands), tumor necrosis factor α (TNF-α, R&D, the Netherlands) concentrations in the culture supernatant were measured by commercial ELISA kits according to the instruction of the manufacturer. GM-CSF, MCP1, and VEGF (all Bio-rad, Veenendaal, the Netherlands) were measured by Magpix (Luminex Corporation). Lactate was measured by a Lactate Fluorometric Assay Kit (Biovision, CA, USA).

### Metabolic assays using the seahorse system

TC-induced macrophages were cultured as described above and after 24 h detached with Versene (ThermoFisher Scientific) from the culture plates. 1 × 10^5^ cells in triplicate were plated onto overnight-calibrated cartridges in assay medium (RPMI with 0.6 mM glutamine, pH adjusted to 7.4. For OCR also 5 mM glucose and 1 mM pyruvate was added) and incubated for 1 h in a non-CO_2_ corrected incubator at 37^°^C. OCR and ECAR were analyzed using a cell mito stress (OCR) or glycolysis stress test (ECAR) kit in a XFp analyzer (Seahorse bioscience, Copenhagen, Denmark) with final concentrations of 1 μM oligomycin, 1 μM FCCP, 0.5 μM Rotenone/Antimycin A, 10 mM glucose, and 50 mM 2-DG.

### Western blot assessments

TC-induced macrophages were cultured as described above and lysed after 24 h being relieved from the tumor cells. Total protein amounts were determined by BCA assay (Thermo Fisher Scientific) and equal amounts of proteins were loaded on pre-casted 4–15% gels (Biorad, CA, USA). The separated proteins were transferred to a nitrocellulose membrane (Biorad), which was blocked in 5% BSA (Sigma). Incubation overnight at 4^°^C with rabbit polyclonal antibodies against p-S6K, p-4EBP1, p-mTOR (Cell Signaling, Danvers, MA, USA), and actin (Sigma) were used to determine the protein expression, which was visualized using a polyclonal secondary antibody (Dako, Belgium) and SuperSignal West Femto Substrate (Thermo Fisher Scientific) or ECL (Biorad).

### Chromatin immunoprecipitation (ChIP)

Cells were isolated and cultured as described above. After being relieved 24 h from the TPC1 cells, TC-induced macrophages were detached from the plate with 500 μL Trypsin per well and fixed in methanol-free 1% formaldehyde and stored at 4°C until further processing. Fixed cells were sonicated for 10 min in a Bioruptor pico sonicator (Diagenode, Seraing, Belgium). Immunoprecipitation was performed using antibodies against H3K4me3 and H3K9me3 (Diagenode, Seraing, Belgium). After ChIP, DNA was processed further for qPCR analysis using the SYBR green method. Samples were analyzed by a comparative Ct method. Primer sequences can be found in S1 Appendix.

### Immunohistochemistry

Glycolytic enzymes third human isoform of 6-phosphofructo-2-kinase/fructose-2,6-bisphosphatase and pyruvate kinase 2 (PFKFB3, PKM2), lactate receptor GPR81 and CD68 protein expression was evaluated by immunohistochemical staining of formalin-fixed paraffin-embedded (FFPE) TC tissue sections. To remove the paraffin, tissues were incubated twice in xylene and successively in 100%, 96%, and 70% of alcohol for 5 min each step. Antigens were retrieved with citrate buffer for 2 min in the microwave (800 W) and 10 minutes at RT (Citrate buffer: pH = 6.0, 16.4 mL sodium citrate (0.1 M) with 3.6 mL citric acid (0.1 M) in 180 mL H_2_O). The endogenous peroxidase activity was blocked with 3% of H_2_O_2_ in methanol for 15 min at RT. Furthermore, since tumor-like tissues contain endogenous biotin, this was blocked in the tissue sections by an avidin/biotin blocking kit according to the manufacturers' protocol (Vector Laboratories, CA, USA). Sections were incubated with 20% goat serum diluted in PBS for 10 min and subsequently with the first antibody (PFKFB3 (ab96699, Abcam, 1:100), PKM2 (3198, Cell Signaling, 1:100), GPR81 (ab188647, Abcam, 1:50), CD68 (KP1, DAKO, 1:8000) all diluted in PBS supplemented with 5% goat/rabbit serum, overnight at 4°C. After washing with PBS, sections were incubated with a second HRP-conjugated antibody 1:200 diluted in PBS for 1 h at RT. The ABC-HRP complex (ABCkit-HRP Vector PK-6101), 1:500 diluted in PBS, was applied to the sections for 30 min at RT. The substrate solution was added for 7 min at RT: 0.5 ml of DAB in 9.5 mL of PBS and 10 μL of H_2_O_2_. Tissues were counterstained with haematoxylin for 30 sec at RT. Slides were dehydrated with consecutive incubation in 70%, 96%, 100% of alcohol and xylene (two times) for 5′ each step. Sections were mounted in Permount.

### Statistical analysis

Differences in cytokine, lactate, VEGF production, trimetylation, and phosphorylation were analyzed using Wilcoxon signed-rank test. All analyses were performed in Graphpad prism 5 (CA, USA). *p < 0.05, **p < 0.01. Data are shown as means ± SEM. Information on additional statistical analyses can be found in the supplementary data.

## Supplementary Material

KONI_A_1229725_supplementary_data.zip
