# Supplementary material for: Transcriptional and metabolic reprogramming induce an inflammatory phenotype in non-medullary thyroid carcinoma-induced macrophages
Source: Oncoimmunology. 2016 Sep 9;5(12):e1229725. doi: 10.1080/2162402X.2016.1229725 (PMC5213309; doi:10.1080/2162402X.2016.1229725)
Supplement: KONI_A_1229725_supplementary_data.zip [file koni-05-12-1229725-s001.zip › KONI_A_1229725_s02.docx]

**Supplementary data Arts et al.**

1. Supplementary Experimental procedures
2. Supplementary Figures
3. Supplementary Tables
4. Supplementary References
5. **Supplementary experimental procedures**
   1. ***Preprocessing of sequenced data***

All reads were aligned against the human hg19 reference genome by TopHat2 v2.0.11 ^1^ using default parameters. The data was imported into Partek Genomics Suite v6.6 (PGS) to deduct gene and transcript information before performing normalization using statistical software R (v3.0.2) and the DESeq2 package (<http://dx.doi.org/10.1101/002832>). Normalized read counts were floored to a value of at least 1 after correcting for donor introduced variance. Subsequent to flooring, the dataset was trimmed by defining a gene as expressed if the maximum value over all group means was higher than 10.

***Identification of differentially expressed genes***

To calculate the top variable genes (TPC1 vs. RPMI) and differentially expressed (DE) genes within the dataset between TC-induced macrophages and RPMI controls (4-hr and 24-hr) a two-way ANalysis Of Variance (ANOVA) was performed using PGS. Genes were defined to be DE when having a fold change (FC) of ≥ 1.5 and an unadjusted p-value of ≤ 0.05. Based on the ANOVA model, Hierarchical clustering (HC) was performed on the top 1000 variable genes within the dataset (TPC1 vs. RPMI) using default settings in PGS to observe the transcriptome similarity between the samples on a more detailed level.

***Gene set enrichment analysis***

Gene Set Enrichment Analysis (GSEA) was performed by PGS using 10,000 permutations utilizing the hallmark and reactome pathway gene-sets (<http://software.broadinstitute.org/gsea/msigdb/index.jsp>) to find enriched pathways in TPC1 co-cultured macrophages (4-hr and 24-hr, compared to RPMI controls). To identify to which polarization state the TC-induced macrophages relate the most, GSEA was also performed utilizing 49 gene sets reflecting a continuum of macrophages activation-states ^2^. All GSEA results were visualized by volcano plots.

***Generation of a co-regulation network based on the DE genes among conditions***

In order to identify similarities between gene expression throughout all conditions (n=16), we employed co-regulation analysis (CRA) based on Pearson correlation coefficients (≥ 0.70) by using BioLayout Express3D ^3^ on the top 500 variable genes within the dataset (TPC1 vs. RPMI). For visualization purposes, the obtained network was imported in Cytoscape (<http://www.cytoscape.org/>). To identify subnetworks specific for the conditions within the dataset, the FC over mean of all conditions was computed for each condition and subsequently mapped onto the CRA network. As validation to this approach, using the mean expression values, Self-Organizing Map (SOM) clustering (map height: 10 by 10) was performed in PGS to identify condition specificity of genes. To validate GSEA results, all PI3K/AKT/MTOR- and glycolysis-related gene lists were extracted from hallmark and reactome pathway gene sets (<http://software.broadinstitute.org/gsea/msigdb/index.jsp>). These genes were subsequently mapped onto the CRA network to identify condition specificity for these pathways. Transcriptional regulators within the dataset were identified using a TF list ^4^ and subsequently mapped onto the CRA network.

***Gene ontology enrichment analysis and GO network visualization***

To link condition-specific genes to prior knowledge, we applied Gene Ontology Enrichment Analysis (GOEA) on a gene set specific (FC condition vs. mean all conditions: ≥ 1; present in CRA network) for the TPC1 co-cultured macrophages (24-hr). Subsequently, to visualize the data we employed the BiNGO ^5^, EnrichmentMap ^6^, and Word Clouding ^7^ plug-ins in Cytoscape.

- 1. ***Primer sequences used for ChIP experiments***

Primer sequences for ChIP experiments

|  | Forward | Reverse |
| --- | --- | --- |
| myoglobulin (positive control H3K4me3) | AGCATGGTGCCACTGTGCT | GGCTTAATCTCTGCCTCATGAT |
| H2B (negative control H3K4me3) | TGTACTTGGTGACGGCCTTA | CATTACAACAAGCGCTCGAC |
| TNF | GTGCTTGTTCCTCAGCCTCT | ATCACTCCAAAGTGCAGCAG |
| IL6 | AGGGAGAGCCAGAACACAGA | GAGTTTCCTCTGACTCCATCG |
| ZFN274_3UTR (positive control H3K9me3) | AAGCACTTTGACAACCGTGA | GGAGGAATTTTGTGGAGCAA |
| GAPDH (negative control H3K9me3) | CACCGTCAAGGCTGAGAACG | ATACCCAAGGGAGCCACACC |

1. **Supplementary Figures**

***Supplementary Fig. S1.*** **Transcriptome analysis of TC-induced macrophages**. (A) Scheme outlining DE gene numbers derived from various comparisons. Arrow indicates direction of differential expression (upregulated, red; downregulated, blue; FC ≥ 1.5, unadjusted p-value ≤ 0.05). (B) For each gene within the dataset, condition specificity is computed applying SOM clustering and subsequently mapped onto the CRA network. (C) Volcano plots of normalized enrichment scores (NES) and enrichment p-values based on GSEA using reactome pathway gene sets (MSigDB, Broad Institute). Data are shown for TPC1 co-cultured macrophages (4-hr and 24-hr). Dark (NES ≥ 1; p value ≤ 0.05) and light (NES ≥ 1; p value > 0.05, ≤ 0.1) red circles show gene sets positively enriched. Dark and light blue circles show gene sets depleted (NES ≤ -1). (D) Transcriptional regulators are mapped onto the CRA network (yellow and orange node(s)). (E) Volcano plots of normalized enrichment scores (NES) and enrichment p-values based on GSEA using stimuli-specific gene sets ^2^. Data are shown for TPC1 co-cultured macrophages (4-hr and 24-hr). Red circles (NES ≥ 1; p value ≤ 0.05) show gene sets positively enriched. Blue circles show gene sets depleted (NES ≤ -1).


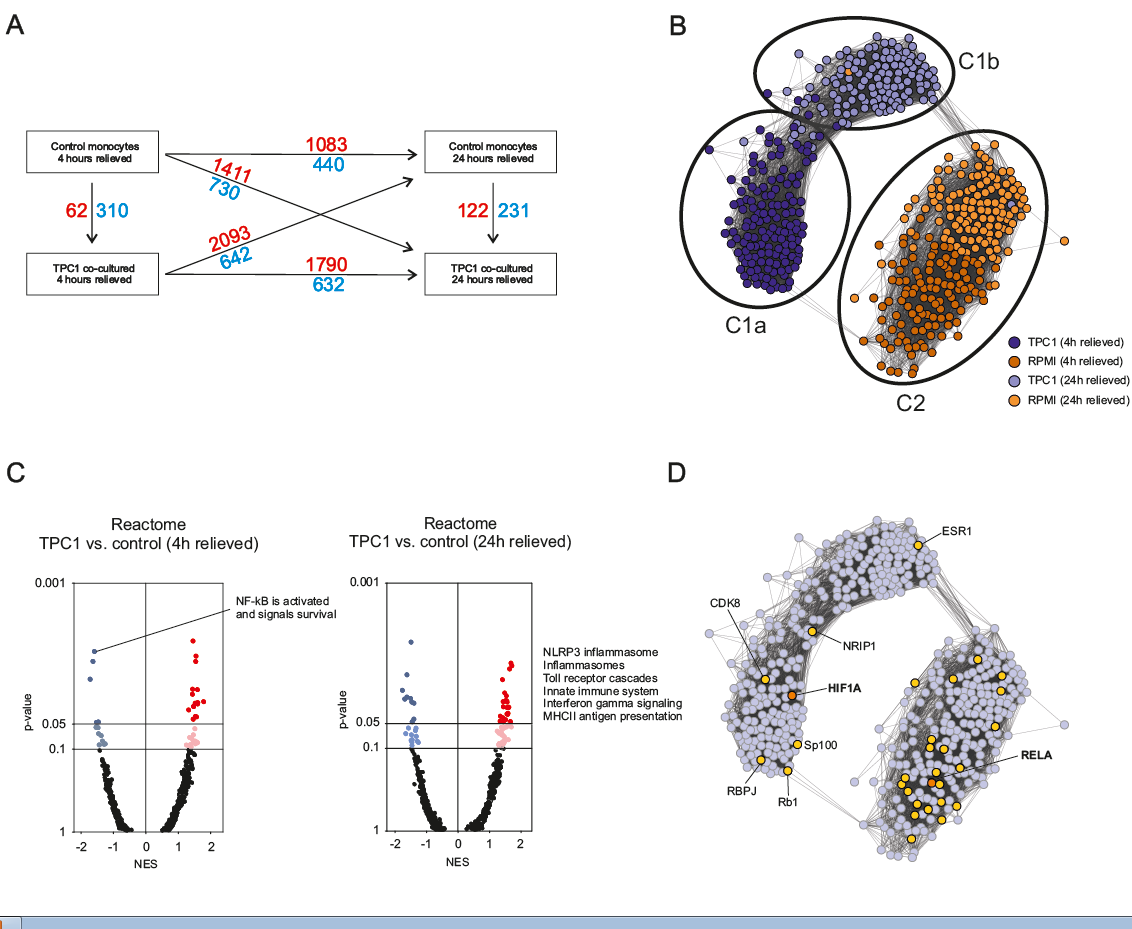


***
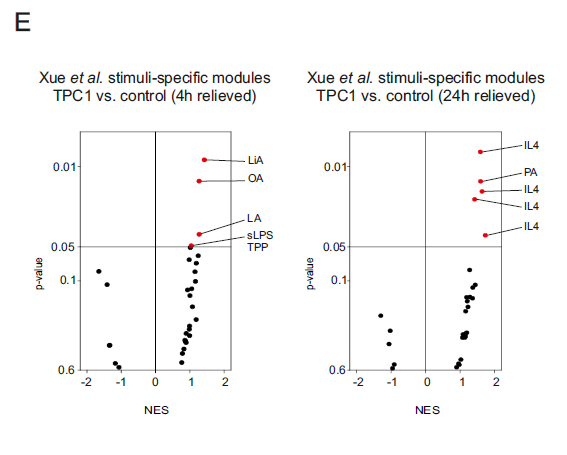
***

***Supplementary Fig. S2.*** **TC-induced macrophages have a proinflammatory phenotype.** (A) Monocytes were incubated for 24h with thyroid cancer cell lines in a trans-well system and restimuled with RPMI, P3C or IL-1α for 24h. (B) In the LPS restimulated TC-induced macrophages, also IL-8, MCP-1 and GM-CSF concentrations were measured in the culture medium.

***Supplementary Fig. S3.* A VEGF antagonist does not reduce the proinflammatory phenotype.** A VEGF receptor antagonist was added to the culture system during coculture with TPC1 tumor cells. After 24h coculture, macropaghes were restimulated with LPS and TNF and IL-6 production was determined in the supernatants.

***Supplementary Fig. S4.* Intracellular Acetyl CoA and glutamate.**  Monocytes were incubated for 24h with TPC1 thyroid cancer cells in a trans-well system. After being relieved from the TPC1 cells for 24h, macrophages were lysed and intracellular concentrations of Acetyl CoA and glutamate were determined.

***Supplementary Fig. S5.* Inhibition of metabolic pathways, TNF results.** Monocytes were incubated for 24h with TPC1 thyroid cancer cells in a trans-well system with or without inhibitors of mTOR (rapamycin), the pentose phosphate pathway (6-AN), glutamine metabolism (BPTES), β-oxidation of fatty acids (etomoxir), or the electron transport chain complex V (oligomycin). After being relieved from the TPC1 cells and inhibitors for 24h, were restimulated with LPS and TNF production was determined.

**Supplementary Fig. S6. Metabolism of TC-induced macrophages is changed. (**A) Immunohistochemical analysis of PFKFB3, PKM2 and GPR81 and their colocalization with CD68 positive TAMs in differentiated TC. Results are representative of stained FFPE tissue material derived from five patients. (B) Immunohistochemical analysis CD68 positive macrophages in healthy thyroid tissue. Hardly any positively stained cells can be found. 200x magnification.


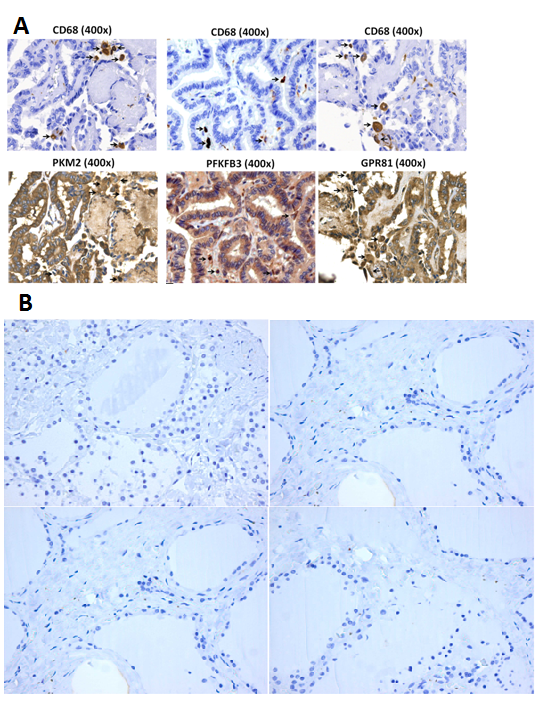


1. **Supplementary Tables**

Table S1. Differentially expressed genes between TC-induced macrophages and RPMI control macrophages.

Table S2. Tabular representation of Supplementary Fig. S1C.

Table S3. Tabular representation of Fig. 1E.

1. **Supplementary References**

1. Kim D, Pertea G, Trapnell C, Pimentel H, Kelley R, Salzberg SL. TopHat2: accurate alignment of transcriptomes in the presence of insertions, deletions and gene fusions. Genome biology 2013; 14:R36.

2. Xue J, Schmidt SV, Sander J, Draffehn A, Krebs W, Quester I, De Nardo D, Gohel TD, Emde M, Schmidleithner L, et al. Transcriptome-based network analysis reveals a spectrum model of human macrophage activation. Immunity 2014; 40:274-88.

3. Theocharidis A, van Dongen S, Enright AJ, Freeman TC. Network visualization and analysis of gene expression data using BioLayout Express(3D). Nature protocols 2009; 4:1535-50.

4. Fulton DL, Sundararajan S, Badis G, Hughes TR, Wasserman WW, Roach JC, Sladek R. TFCat: the curated catalog of mouse and human transcription factors. Genome biology 2009; 10:R29.

5. Maere S, Heymans K, Kuiper M. BiNGO: a Cytoscape plugin to assess overrepresentation of gene ontology categories in biological networks. Bioinformatics 2005; 21:3448-9.

6. Merico D, Isserlin R, Stueker O, Emili A, Bader GD. Enrichment map: a network-based method for gene-set enrichment visualization and interpretation. PloS one 2010; 5:e13984.

7. Oesper L, Merico D, Isserlin R, Bader GD. WordCloud: a Cytoscape plugin to create a visual semantic summary of networks. Source code for biology and medicine 2011; 6:7.
